# The PCOS‐to‐PMOS shift in the broader history of nomenclature changes and its uncertain impact on clinical practice

**DOI:** 10.1111/aogs.70380

**Published:** 2026-10-29

**Authors:** Elina Aleksejeva‐Zagura, Piotr Laudański, Ganesh Acharya, Andres Salumets

**Affiliations:** ^1^ Institute of Genomics University of Tartu Tartu Estonia; ^2^ Institute of Veterinary Medicine and Animal Sciences Estonian University of Life Sciences Tartu Estonia; ^3^ Department of Obstetrics, Gynecology and Gynecological Oncology Medical University of Warsaw Poland; ^4^ Women's Health Research Institute Calisia University Kalisz Poland; ^5^ OVIklinika Infertility Center Warsaw Poland; ^6^ Center for Fetal Medicine Karolinska University Hospital Stockholm Sweden; ^7^ Division of Obstetrics and Gynaecology, Department of Clinical Science, Intervention and Technology (CLINTEC), Karolinska Institutet Karolinska University Hospital Stockholm Sweden; ^8^ Department of Obstetrics and Gynecology Institute of Clinical Medicine, University of Tartu Tartu Estonia; ^9^ Celvia CC AS Tartu Estonia

**Keywords:** Healthcare economics, PCOS (Polycystic ovary syndrome), PMOS (Polyendocrine metabolic ovarian syndrome)

## Abstract

Proposed renaming of polycystic ovary syndrome (PCOS) to polyendocrine metabolic ovarian syndrome (PMOS) aims to broaden multidisciplinary care. However, it may obscure reproductive manifestations and introduce phenotypic ascertainment bias. Historical examples of nomenclature changes suggest that clinical benefits depend heavily on diagnostic, therapeutic, and etiological advances and education. Although formal cost‐effectiveness analysis of nomenclature changes are lacking, our preliminary estimates suggest that implementation may entail substantial cost and healthcare resource requirements, while its clinical benefits remain uncertain.

## Graphical abstract

Proposed renaming of polycystic ovary syndrome (PCOS) to polyendocrine metabolic ovarian syndrome (PMOS) aims to broaden multidisciplinary care. However, it may obscure reproductive manifestations and introduce phenotypic ascertainment bias. Historical examples of nomenclature changes suggest that clinical benefits depend heavily on diagnostic, therapeutic, and etiological advances and education, whereas formal cost‐effectiveness analysis of renaming cost have not been calculated.

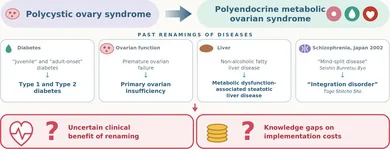

**Abbreviations**
ICD: International classification of disease
PCOS: Polycystic ovary syndrome
PMOS: Polyendocrine metabolic ovarian syndrome
POF: Premature ovarian failure
POI: Primary ovarian insufficiency
QALY: Quality‐adjusted life‐year

**Key message**

Historical examples show that disease nomenclature changes can improve conceptual clarity and reduce stigma, but benefits depend on broader context. Renaming PCOS to PMOS may also carry unintended clinical consequences, including metabolic overemphasis and underrecognition of reproductive manifestations.

Teede and colleagues report on the global consensus process that has suggested renaming polycystic ovary syndrome (PCOS) to polyendocrine metabolic ovarian syndrome (PMOS) across major medical journals including Lancet, Nature Medicine, and Fertility & Sterility.^1^, ^2^, ^3^ The process was strongly informed by patients and patient organizations, who highlighted that the term “polycystic” can be misleading by implying pathological ovarian cysts and that the emphasis on the ovaries does not adequately reflect the condition's broader endocrine, metabolic, reproductive, and other manifestations. Teede et al. argue that the proposed name change will broaden clinical responsibility beyond gynecology, promote multidisciplinary care, attract research investment, reduce patient stigma, and improve recognition and management of associated metabolic, cardiovascular, and reproductive risks. Ultimately, they propose that the new nomenclature will facilitate earlier diagnosis, more comprehensive care, and standardized disease classification globally. Given its implications for clinical practice and disease conceptualization within obstetrics and gynecology, the rationale and limitations of the proposed nomenclature change merit discussion.

We agree that PCOS/PMOS remains frequently diagnosed late, while its etiology is incompletely understood and research investment remains disproportionate to disease burden,^1^, ^4^, ^5^ however, terminology change should not be conflated with improvement in clinical outcomes. The proposed nomenclature change has been subject to several substantive criticisms. One concern is that foregrounding the metabolic aspects may inadvertently attenuate recognition of the syndrome's reproductive and endometrial pathophysiology, potentially reinforcing a conceptualization in which reproductive manifestations are viewed as secondary rather than as integral components of the disorder.^6^, ^7^ The designation “polyendocrine” warrants additional caution, as it may be interpreted as implying dysfunction of multiple endocrine glands, rather than dysregulation across interconnected endocrine pathways.^8^ Furthermore, metabolic framing may introduce phenotypic ascertainment bias, particularly for lean and nonhyperandrogenic presentations that are relatively prevalent in East Asian, Mediterranean, and Middle Eastern populations and may exhibit lower metabolic risk.^9^, ^10^

Importantly, to date, it has not been demonstrated that disease renaming alone leads to measurable improvements in diagnostic rates, treatment outcomes, quality‐adjusted life‐years (QALYs), healthcare efficiency, or research translation. Meanwhile, the implementation costs, budget impact, and incremental economic value of the PMOS transition have not yet been prospectively estimated. Historical examples illustrate that although terminology can improve conceptual clarity, clinical benefits generally arise from advances in diagnostics, management strategies, and therapeutics.

For example, the transition from age‐based categories of “juvenile” and “adult‐onset” diabetes to type 1 and type 2 diabetes, which improved understanding by aligning disease classification with underlying pathophysiology.^11^ However, improvements in patient outcomes are more plausibly attributable to advances in insulin therapy, glucose monitoring, cardiovascular risk management, and disease‐modifying interventions than to the terminology itself.^12^

Similarly, replacing premature ovarian failure (POF) with primary ovarian insufficiency (POI) reduced potentially misleading terminology. Nevertheless, the change itself has not been shown to independently improve clinical outcomes. Despite the adoption of POI terminology in 2008, concerns regarding delayed diagnosis and inadequate clinical management persisted years later.^13^ The subsequent incorporation of POI into international clinical guidelines, including the European Society of Human Reproduction and Embryology guideline in 2014, helped establish the terminology globally; however, educational initiatives persist to improve healthcare providers' awareness and optimize patient care.^14^ Diagnostic challenges remain because POI presents with heterogeneous clinical manifestations, and diagnostic criteria are still not fully harmonized across professional societies.

Another relevant example is metabolic dysfunction‐associated steatotic liver disease. The condition was initially renamed from nonalcoholic fatty liver disease to metabolic dysfunction‐associated fatty liver disease in 2020 to better reflect the relationship between hepatic steatosis and metabolic risk factors such as obesity and type 2 diabetes. The terminology was subsequently revised to metabolic dysfunction‐associated steatotic liver disease to distinguish metabolic liver disease from alcohol‐related liver disease and to provide a more inclusive framework.^15^ Although improvements in QALYs or other direct healthcare outcomes following the name change have not been demonstrated, the terminology shift has been considered clinically meaningful because it moves away from a diagnosis of exclusion toward a positive diagnosis based on metabolic dysfunction; however, this is a qualitative and conceptual consideration.

Interestingly, in Japan, renaming schizophrenia in 2002 from *Seishin Bunretsu Byo* (“mind‐split disease”) to *Togo Shitcho Sho* (“integration disorder”) was initiated based on the request of patients' families group and it increased diagnostic disclosure from 36.7% to 69.7% over 3 years.^16^ This demonstrates that nomenclature effects can be measured rather than assumed. Nonetheless, this improvement coincided with clinician education and revised practice guidelines, suggesting that benefits reflected the broader implementation strategy rather than the terminology alone. Furthermore, this example may be particularly relevant to a highly stigmatized psychiatric disorder, where less stigmatizing terminology may facilitate acceptance and communication of the diagnosis.

Across the nomenclature transitions considered, the independent effects of revised terms on patient outcomes and cost‐effectiveness have not been established because terminology changes have typically coincided with advances in diagnosis, treatment, and care. The renaming of schizophrenia in Japan, which was meant to decrease stigma, did measurably improve diagnostic disclosure.

The proposed PCOS‐to‐PMOS transition is primarily intended to stimulate etiological understanding and emphasize metabolic dysfunction. However, in contrast to examples of name‐changes above, the underlying biology, causal mechanisms, and potential PMOS disease subtypes remain incompletely characterized. Thereby, it remains uncertain how renaming alone would address current gaps in diagnosis, treatment stratification, and symptom management.^5^ Importantly, much of the existing knowledge about the condition has been generated under the term PCOS. This name itself, however, is relatively new as it was adopted in the 60s, whereas it was known before as Stein‐Leventhal syndrome following the 1935 description by Stein and Leventhal.^17^ As the saying goes, “a rose by any other name would smell as sweet”; changing terminology does not automatically change biological understanding or clinical reality.

Implementation would also use healthcare resources; however, no cost‐effectiveness analysis of disease name changes has been done for any of the aforementioned diseases. The proposed plan for PCOS‐to‐PMOS transition includes patient and professional materials, education, textbook and electronic health record updates, work with software vendors, and ICD (international classification of disease) code adoption.^1^ Considering approximately 15 000 hospitals in EU,^18^ an illustrative one‐off cost of €2000 per hospital would equal about €30 million. This is not a formal budget‐impact estimate. Experience with ICD‐10 shows that classification changes can require training, documentation and software updates, testing, and temporary productivity losses.^19^ Furthermore, UK data recorded 0.35 PCOS‐related contacts per diagnosed woman‐year,^20^ supporting consultation volumes in the millions. If 2 extra minutes were required across 10 million consultations in one transition year, this would equal approximately 333 000 clinician‐hours. Because PMOS‐specific data are not available, these figures are only illustrative estimates. Any cost analysis should clearly state its assumptions and the time period covered.^21^ These costs should be balanced against the lack of demonstrated patient benefit from renaming alone.

At the same time, women's health remains substantially underfunded, with only approximately 1% of research and development investment directed toward women‐specific conditions.^4^ This underinvestment contributes to persistent diagnostic delays and gaps in evidence‐based care across multiple areas of women's health. Therefore, interventions with demonstrated capacity to improve outcomes should be prioritized.

## AUTHOR CONTRIBUTIONS

**Elina Aleksejeva‐Zagura:** Conceptualization; writing – original draft. **Piotr Laudański:** Formal analysis; writing – review and editing. **Ganesh Acharya:** Writing – review and editing. **Andres Salumets:** Conceptualization; writing – review and editing. All authors read and approved the final manuscript.

## CONFLICT OF INTEREST STATEMENT

The authors declare no conflicts of interest.

## Data Availability

Data sharing not applicable to this article as no datasets were generated or analysed during the current study.
